# OLA1 contributes to epithelial-mesenchymal transition in lung cancer by modulating the GSK3β/snail/E-cadherin signaling

**DOI:** 10.18632/oncotarget.7224

**Published:** 2016-02-06

**Authors:** Li Bai, Zubin Yu, Jiawei Zhang, Shuai Yuan, Chen Liao, Prince V.S Jeyabal, Valentina Rubio, Huarong Chen, Yafei Li, Zheng-Zheng Shi

**Affiliations:** ^1^ Department of Respiratory Diseases, Xinqiao Hospital, Third Military Medical University, Chongqing, China; ^2^ Department of Translational Imaging, Houston Methodist Research Institute, Houston, Texas, USA; ^3^ Department of Thoracic Surgery, Xinqiao Hospital, Third Military Medical University, Chongqing, China; ^4^ Cancer Institute, The Second Affiliated Hospital, School of Medicine, Zhejiang University, Hangzhou, China; ^5^ Department of Epidemiology, College of Preventive Medicine, Third Military Medical University, Chongqing, China

**Keywords:** Obg-like ATPase 1, epithelial-mesenchymal transition, GSK3 β, E-cadherin, lung cancer

## Abstract

Obg-like ATPase 1 (OLA1) belongs to the Obg family of *P*-loop NTPases, and may serve as a “molecular switch” regulating multiple cellular processes. Aberrant expression of OLA1 has been observed in several human malignancies. However, the role of OLA1 in cancer progression remains poorly understood. In this study, we used the Kaplan-Meier plotter search tool to show that increased expression of OLA1 mRNA was significantly associated with shorter overall survival in lung cancer patients. By immunohistochemical analysis we discovered that levels of OLA1 protein in lung cancer tissues were positively correlated with TNM stage and lymph node metastasis, but negatively correlated with the epithelial-mesenchymal transition (EMT) marker E-cadherin. Knockdown of OLA1 in a lung adenocarcinoma cell line rendered the cells more resistant to TGF- β-induced EMT and the accompanied repression of E-cadherin. Furthermore, our results demonstrated that OLA1 is a GSK3 β-interacting protein and inhibits GSK3 β activity by mediating its Ser9 phosphorylation. During EMT, OLA1 plays an important role in suppressing the GSK3 β-mediated degradation of Snail protein, which in turn promotes downregulation of E-cadherin. These data suggest that OLA1 contributes to EMT by modulating the GSK3 β/Snail/E-cadherin signaling, and its overexpression is associated with clinical progression and poor survival in lung cancer patients.

## INTRODUCTION

Lung cancer is currently the most common cause of cancer-related deaths worldwide. Despite advances in diagnosis and treatment, the overall 5-year survival rate of lung cancer remains at about 15% [1]. Histologically, it can be classified as small-cell lung carcinoma (SCLC) and non-small-cell lung carcinoma (NSCLC)—the latter mainly consists of adenocarcinoma and squamous cell carcinoma and represents 85% of lung cancer cases [2]. For all types of lung cancer, failure of treatment is largely attributed to advanced stage of the disease and metastasis.

Epithelial-mesenchymal transition (EMT) is a biological process that enables epithelial cells to acquire mesenchymal phenotypes, including increased migratory and invasive capabilities [3]. EMT has been implicated in tumorigenesis and progression, especially tumor invasion and metastasis [4, 5]. A critical molecular event in EMT is the Snail-mediated down-regulation of the epithelial marker E-cadherin, which results in disruption of cell-to-cell adhesion and subsequent acquisition of more migratory and invasive phenotype. Snail binds to the E-box sequence in the promoter of the E-cadherin gene and represses transcription of E-cadherin [6, 7]. Loss of E-cadherin has been found to correlate with low histologic grade, advanced stage and poor prognosis in many epithelial tumors including lung cancer [8, 9].

OLA1 (Obg-like ATPase 1) belongs to the translation-factor-related (TRAFAC) class, the Obg family, and the YchF subfamily of *P-*loop GTPases [10, 11]. *P*-loop GTPases are the most abundant nucleotide-binding proteins, and are involved in the regulation of diverse cellular processes, including protein translation, intracellular transport, signal transduction, cell proliferation, and stress response [12, 13]. The OLA1/YchF proteins are highly conserved from bacteria to humans, and unlike other Obg family members, they bind and hydrolyze both ATP and GTP [11, 14]. Our group demonstrated that human OLA1 functions as an intrinsic regulator in cellular stress responses such as oxidative stress [15] and heat shock [16], and down-regulation of OLA1 causes changes in cell migration and invasiveness in cultured human breast cancer cells [17]. Recent study from other group has also shown OLA1 overexpressed in multiple human malignancies and down-regulated under DNA damage stresses [18].

In the present study, we aimed to investigate the clinical relevance of OLA1 in lung cancer and the underlying mechanisms. We found that expression of OLA1 was negatively correlated with E-cadherin, the EMT marker, in lung cancer tissues, and high-level expression of OLA1 was associated with more advanced TNM stages, lymph node metastasis and poor prognosis. The study also showed that OLA1 is a GSK3β-interacting protein, and knockdown of OLA1 causes an under-phosphorylated yet hyperactive GSK3β, promoting degradation of its substrate Snail, and thus preserving the expression of E-cadherin. We conclude that OLA1 contributes to the EMT of lung cancer cells through regulating the GSK3β/Snail/E-cadherin signaling pathway.

## RESULTS

### OLA1 expression correlates with clinicopathological parameters of lung cancer

**Figure 1:**
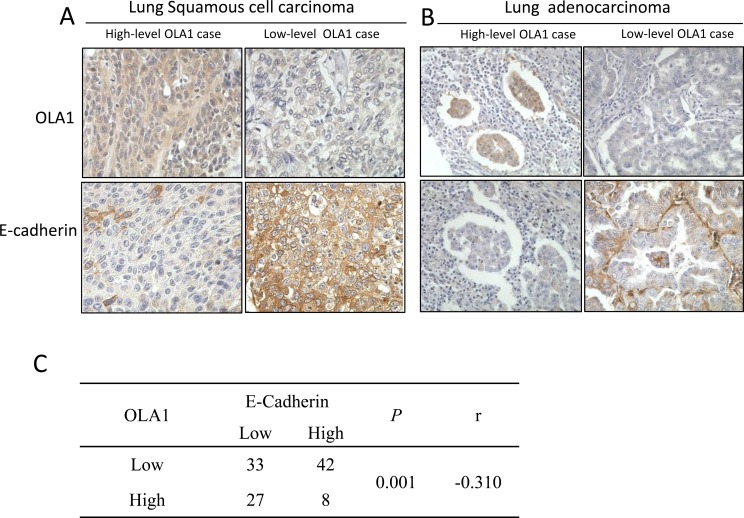
IHC analysis of OLA1 and E-cadherin expression in human lung cancer tissues Comparison of OLA1 and E-cadherin expression within the same tumor tissue by pairing the microphotographs (vertically) taken from the nearby serial sections (200×original magnification). The left two columns are from two cases of squamous cell carcinoma (**A**) and the right two columns are from two cases of adenocarcinoma (**B**) Note that the levels of OLA1 staining are largely opposite to the levels of E-cadherin staining. The result of a Spearman's rank correlation test for correlation between OLA1 and E-cadherin expressions among 110 cases of lung cancer is shown in **C.**

To explore the clinical implication of OLA1 in human lung cancer, we performed immunohisochemical (IHC) analysis with a lung tissue microarray. Of the 110 cancer samples, 83 were positive for OLA1 expression in the cytoplasm (Figure 1), whereas none of the 10 normal lung tissues showed detectable OLA1 (*p* < 0.0001). Table 1 summarizes the correlation of OLA1 expression with clinicopathological parameters for all the cancer cases. The frequency of high-expression OLA1 was significantly higher in squamous cell carcinoma (56.1%, 23 of 41) than that in other histological types (17.4%, 12 of 69) (*p* < 0.0001). The rate of high-expression OLA1 in tumors of TNM stage III and IV (48.5%,16 of 33) was significantly higher than that in tumors of stage I (16.7%,8 of 48) and II (37.9%, 11 of 29) (*p* = 0.007). Moreover, high-expression OLA1 was found more often in cases with lymph node metastasis than in cases without lymph node metastasis (42.9% vs.20.4%, *p* = 0.01). However, no significant association was observed between OLA1 levels and differential grade, sex, and age. We further evaluated the OLA1 expression in larger tissue sections cut from formalin-fixed paraffin-embedded tumor and paired normal lung tissues from 10 patients with lung adenocarcinoma. IHC analysis showed that OLA1 expression was lower in normal lung tissues than in paired tumor tissues, and low-expression OLA1 was mainly localized in the cytoplasm of the bronchial and alveolar epithelium (data not shown).

**Table 1:** Correlation of OLA1 and E-cadherin expression with clinicopathological parameters in lung cancer tissues

		OLA1			E-Cadherin		
	Total	Low (%)	High (%)	P^*^	Low (%)	High (%)	P^*^
**Sex**							
Male	75	47(62.7)	28(37.3)	0.069	43 (57.3)	32 (42.7)	0.390
Female	35	28(80.0)	7(0.2)		17 (48.6)	18 (51.4)	
**Age (years old)**							
<65	83	59(71.1)	24(28.9)	0.252	48 (57.8)	35 (42.2)	0.225
≥65	27	16(59.3)	11(40.7)		12 (44.4)	15 (55.6)
**Histology**							
Adenocarcinoma	42	33(78.6)	9(21.4)	***<0.0001***	17 (40.5)	25 (59.5)	***0.028***
Squamous cell carcinoma	41	18(43.9)	23(56.1)		24 (58.5)	17 (41.5)	
Small cell carcinoma	6	5(83.3)	1(16.7)		6 (100.0)	0 (0.0)	
Others	21	19(90.5)	2(9.5)		13 (61.9)	8 (38.1)	
**TNM stage**							
I	48	40(83.3)	8(16.7)	***0.007***	21 (43.7)	27 (56.3)	0.125
II	29	18 (62.1)	11 (37.9)		19 (65.5)	10 (34.5)	
III and IV	33	17 (51.5)	16 (48.5)		20 (60.6)	13 (39.4)	
**Grade**							
Well-differentiated	16	10 (62.5)	6 (37.5)	0.918	7 (43.8)	9 (56.3)	0.784
Moderately Differentiated	49	31 (63.3)	18 (36.7)		26 (53.1)	23 (46.9)	
Poorly differentiated	19	11 (57.9)	8 (42.1)		9 (47.4)	10 (52.6)	
**Lymph node metastasis**							
No	54	43 (79.6)	11 (20.4)	***0.010***	24 (44.4)	30 (55.6)	***0.037***
Yes	56	32 (57.1)	24 (42.9)		36 (64.3)	20 (35.7)	

* Chi-square test or Fisher's exact test.

TNM, tumor-node-metastasis.

Next, we performed IHC staining for the EMT marker E-cadherin with tissue sections made from the same-tissue array and analyzed the correlation of OLA1 with E-cadherin among all 110 cancer cases. The incidence of low-expression E-cadherin was higher in cases with lymph node metastasis (64.3%, 36 of 56) than in cases without lymph node metastasis (44.4%, 24 of 54) (*p* = 0.037). We compared the staining of OLA1 and E-cadherin among all 110 cancer cases, and found that the OLA1 levels were inversely correlated with the levels of E-cadherin within the same tumor tissue for all histological types of lung cancer (Figure 1A and 1B). Indeed, when the Spearman's rank correlation test was applied, a significant negative correlation was established (r = −0.310, *p* = 0.001) (Figure 1C).

**Figure 2:**
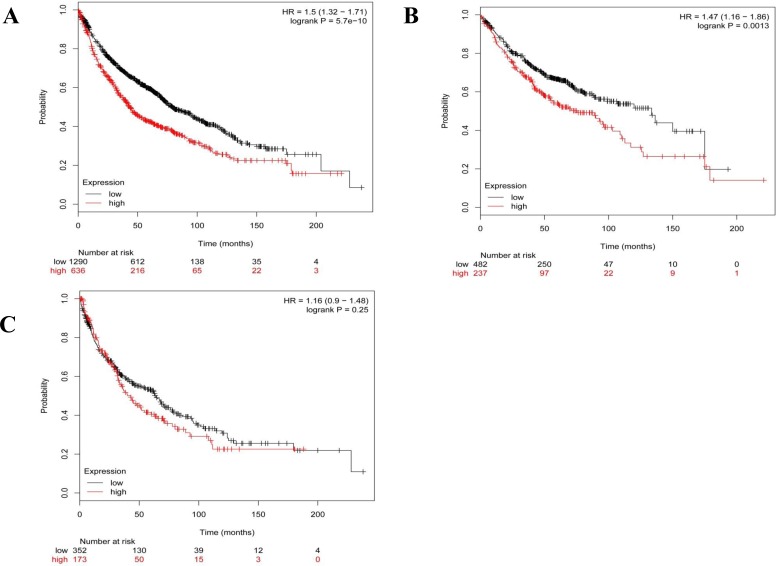
The association of OLA1 mRNA expression with overall survival in patients with lung cancer The Kaplan-Meier plots were generated by selecting the OLA1 probe (219293_s_at) and the survival of all lung cancer patients (**A**), and patients with lung adenocarcinoma (**B**) or squamous carcinoma patients (**C**). The x-axis indicates the time of follow-up, and the y-axis indicates survival probability. Small vertical tick-marks indicate individual patients whose survival times have been right-censored.

However, this lung cancer tissue microarray was not provided with the patients' prognostic information. In order to establish the association between OLA1 expression status and overall survival (OS) in patients with lung cancer, we did a meta-analysis of OLA1 expression among 1928 lung cancer patients using the KM plotter software. We observed that higher expression of OLA1 was significantly associated with shorter OS in lung cancer patients (HR = 1.5; 95% CI = 1.32-1.71; *p* < 0.001, Figure 2A). We also carried out a histological subtype-specific analysis, and found that high-level expression OLA1 was significantly associated with poor OS in lung adenocarcinoma patients (HR = 1.47; 95% CI = 1.16-1.86; *p* = 0.0013, Figure 2B), but not in squamous carcinoma patients (HR = 1.16; 95% CI = 0.9-1.48; *p* = 0.25, Figure 2C).

### OLA1-knockown cells are more resistant to TGF-β1-induced EMT

**Figure 3:**
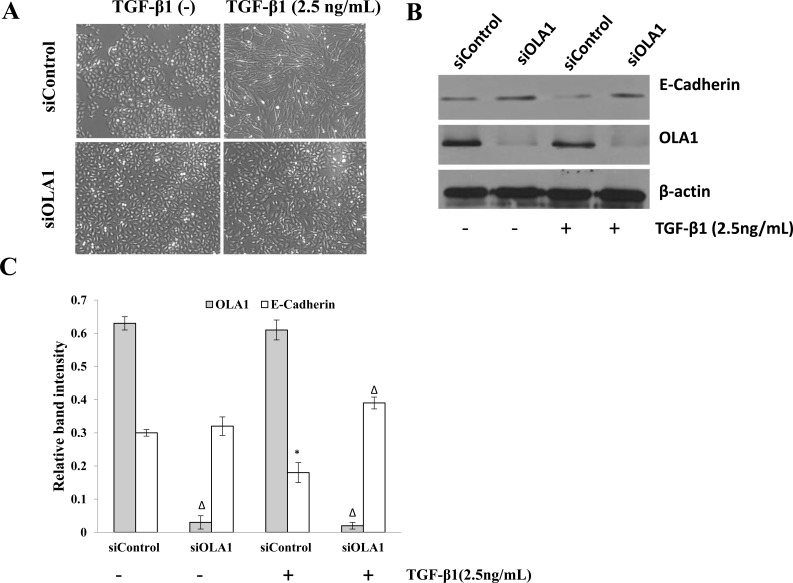
Knockdown of OLA1 results in attenuation of TGF-β1-induced EMT in A549 cells **A.** A549 Cells were transfected with OLA1 siRNA (siOLA1) and control siRNA (siControl) for 48 hours, and then incubated with 2.5 ng/mL TGF-β1 or without TGF-β1 (−) in DMEM containing 5% FBS for 72 hours. Cell images were taken under an inverted light microscope (200×original magnification). **B.** Cell lysates were subjected to Western blot analysis with antibodies as indicated. β-actin was used as a loading control. **C.** Densitometric analysis of Western blots obtained in **B.**, showing band intensities of OLA1 and E-cadherin normalized by that of β-actin. Data represent mean ± SD values from 3 independent experiments. * *p* < 0.01, as compared with the siControl group without the treatment of TGF-β1.^Δ^*p* < 0.01, as compared with the Control siRNA group with or without the treatment with TGF-β1.

The human lung adenocarcinoma cell line A549 was used as the *in vitro* model of EMT in this study. It has been previously reported that TGF-β1 can induce A549 cells to undergo EMT [19-21]. In order to elucidate the role of OLA1 in EMT, we down-regulated the expression of OLA1 in A549 cells by siRNA-mediated gene silencing. We determined that the knockdown of OLA1 protein, although transient, was effective for 1-5 days after the siRNA transfection, thus allowing sufficient time for TGF-β1 treatment. After three-day treatment with 2.5 ng/mL TGF-β1, the control-siRNA transfected A549 cells showed a spindle fibroblast-like morphology with reduced cell-cell contact. In contrast, OLA1-knockdown A549 cells retained their epithelial shape and were arranged in a cobblestone pattern when confluent (Figure 3A), indicating a significant attenuation of TGF-β1-induced EMT in the OLA1-knockdown cells. Immunoblot analysis demonstrated that the observed morphological change in the control cells was accompanied by down-regulation of epithelial marker E-cadherin expression (Figure 3B and 3C). However, no significant decrease of E-cadherin was observed in the OLA1-knockdown cells (Figure 3B and 3C). These data suggested that knockdown of endogenous OLA1 resulted in a resistance to TGF-β1-induced EMT and reduction of E-cadherin expression.

### OLA1-knockdown cells have an altered GSK3β/Snail/E-cadherin pathway

**Figure 4:**
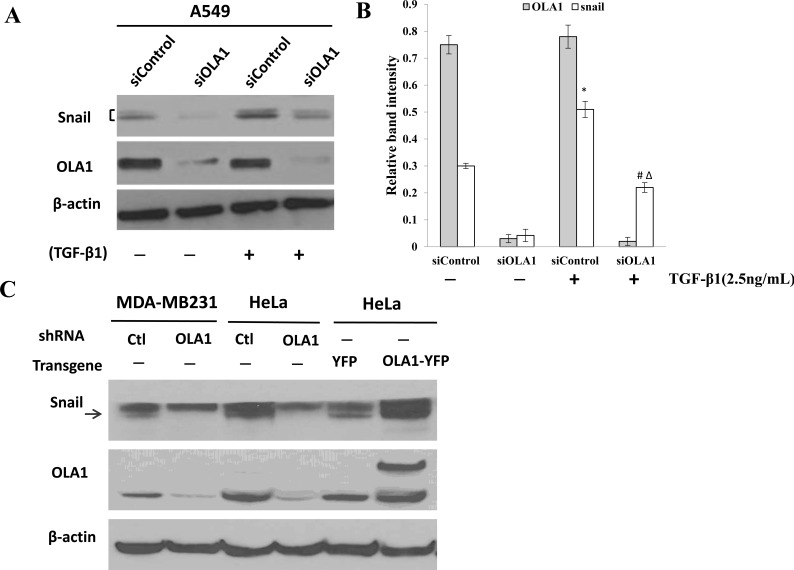
Regulation of Snail protein by OLA1 **A.** Induction of Snail by TGF-β1 in OLA1-knockdown A549 cells. Cells were transfected with control siRNA (siControl) and OLA1 siRNA (siOLA1) for 48 hours, and then treated with TGF-β1 (2.5 ng/mL) or without TGF-β1 (−) for 72 hours. Cell lysates were subjected to Western blot analysis using anti-Snail and anti-OLA1 antibodies. β-actin was used as a loading control. **B.** A quantitative analysis of the relative band intensities for Snail is shown. The bars represent mean ± SD values (*n* = 3).*p < 0.01, as compared with the siControl group without the treatment of TGF-β1; ^#^*p* < 0.01, as compared with the siControl group treated with TGF-β1; ^Δ^*p* < 0.01, as compared with the siOLA1 group without the treatment of TGF-β1. **C.** Basal levels of Snail in MDA-MB-231 and HeLa cells with manipulated OLA1 expression. MDA-MB-231 and HeLa cells were stably transfected with a lentiviral vector containing control (Ctl) shRNA or OLA1-specific shRNA (OLA1). Additionally, HeLa cells were transiently transfected with OLA1-YFP and the control (YFP) vectors. The ectopic expression of OLA1 was confirmed by the presence of a higher molecular weight OLA1-YFP band on top of the endogenous OLA1 band. The Western blot was probed with anti-Snail antibody, and re-probed with anti-OLA antibody (to confirm the gene manipulations) and β-actin antibody (as a loading control). Note that a non-specific band is above the arrow-indicated Snail.

TGF-β1-induced EMT is thought to be mediated by induction of transcriptional repressors, including, most notably, the E-cadherin repressor Snail [22, 23]. We thus measured the levels of Snail in the OLA1-knockdown A549 cells. The basal level of Snail significantly decreased in the OLA1-knockdown A549 cells treated with or without TGF-β1 for three days (Figure 4A). As expected, TGF-β1 up-regulated the Snail protein in both control siRNA- and OLA1 siRNA-transfected cells; however, the Snail protein remained significantly lower in OLA1 siRNA-transfected cells than in control siRNA-transfected cells (Figure 4B).

In order to demonstrate that OLA1-knockdown has a common effect on steady state levels of Snail, we examined Snail in other cell lines in which OLA1 was manipulated with either RNAi or transgene expression. As shown in Figure 4C, MDA-MB-231 and HeLa cells stably transfected with an OLA1-specific shRNA lentiviral vector exhibited markedly decreased Snail compared with the control shRNA-transfected cells. Conversely, HeLa cells transiently overexpressing OLA1 exhibited an increased level of Snail. Taken together, these data strongly suggested that knockdown of OLA1 reversed TGF-β1-induced EMT by downregulating Snail expression.

**Figure 5:**
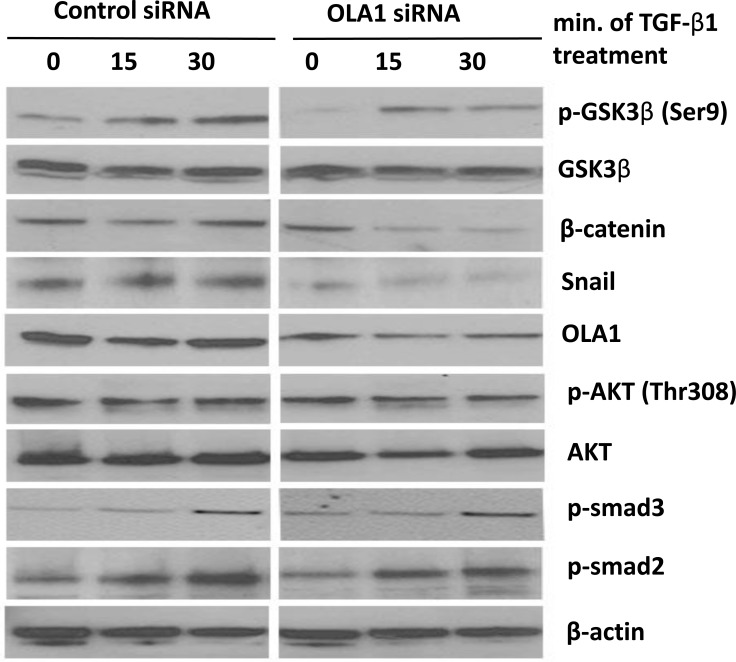
Knockdown of OLA1 inhibited EMT by regulating GSK3β/Snail pathway Cells were transiently transfected with the control siRNA or OLA1 siRNA, and at 48 hours post-transfection the cells were treated with 2.5 ng/mL TGF-β1. Cell lysates collected at indicated time points (0, 15 min, 30 min after treatment with TGF-β1) were subjected to immunoblotting for the levels of GSK3β total protein and its phosphorylation at Ser9 residue. The efficiency of OLA1-knockdown was evaluated by anti-OLA1 probing. Immunoblotting analysis of Akt, phospho-Akt (Thr308), phospho-smad2 (Ser465/467), phospho-smad3 (Ser423/425), β-catenin and Snail was also done. The blot was re-probed with anti-β-actin antibody for equal loading.

Because Snail is a short half-life protein and its stability is primarily regulated by GSK3β [6, 7], we determined whether the observed decrease in Snail protein in OLA1-knockdown cells was regulated by GSK3β activity. We explored the phosphorylation of GSK3β at Ser9 residue, an indicator for inactive state of the kinase, and found that OLA1-knockdown A549 cells had a decreased basal Ser9 phosphorylation (but not the total protein) as compared with the control cells (Figure 5, “0 min”). Treatment of TGF-β1-induced Ser9 phosphorylation in control cells in a time-response manner (from 0 to 30 minutes); however, in the OLA1-knockdown cells the dynamics were markedly attenuated in the first 30 minutes. These data suggested that in OLA1-knockdown cells, GSK3β maintained active state in dephosphorylated form, which was consistent with decreased Snail and increased E-cadherin level.

TGF-β1 is known to signal through two major pathways: the canonical Smad-dependent pathway, and the alternative Smad-independent pathways, including PI3K/Akt signaling [24]. We further examined the phosphorylation status of Smad2/3 and Akt following the 30-minute TGF-β1 treatment. No significant difference in the phosphorylation of these signaling molecules was detected between the OLA1-knockdown and control siRNA-transfected A549 cells (Figure 5). Importantly, two downstream substrates of GSK3β—Snail as well as β-catenin—were found to be diminished in the OLA1-knockdown cells (Figure 4 and Figure 5), further supporting a hyperactive state of GSK3β even after treatment with TGF-β1. Taken together, we speculated that OLA1 regulated EMT by a GSK3β-dependent signal pathway.

### OLA1 and GSK3β are specific interaction partners

**Figure 6:**
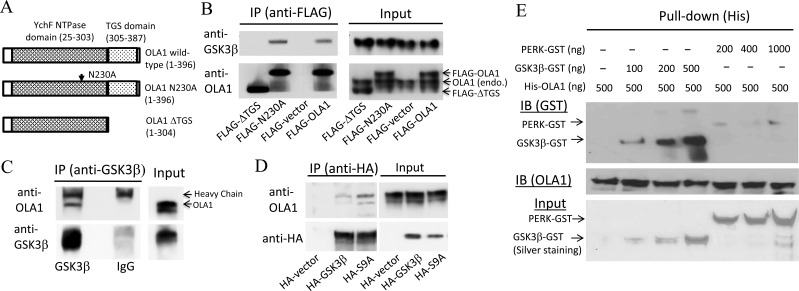
Interaction of OLA1 and GSK3β in intact cells and *in vitro* **A.** Schematic diagrams of the OLA1-related constructs used in this study for ectopic expression of the wild-type OLA1, the full-length OLA1 containing a point mutation (N230A), and the C-terminal deletion mutant OLA1 (∆TGS). **B.** HEK293 cells transiently transfected with FLAG-OLA1, N230A, and FLAG-∆TGS constructs were immunoprecipitated using anti-FLAG-M2 beads. The IP complexes were evaluated by immunoblotting with anti-GSK3β and OLA1 antibodies. **C.** The protein extracts from non-treated HEK293 cells were immunoprecipitated with anti-GSK3β antibody or the control IgG, and the IP products were immunoblotted with anti-OLA1 and GSK3β antibodies. **D.** HEK293 cells transiently transfected with the HA-GSK3β and HA-GSK3β(S9A) constructs expressing the wild-type and the constitutively active mutant GSK3β proteins were immunoprecipitated with anti-HA antibody, followed by immunoblotting with anti-OLA1 and HA antibodies. **E.** Binding of OLA1 with GSK3β *in vitro*. Recombinant His-tagged OLA1 protein (500 μg) was incubated with various amounts of GST-tagged GSK3β or the negative control GST-tagged PERK (protein kinase-like endoplasmic reticulum kinase). His-pull down assays were performed followed by immunoblotting analysis using anti-GST antibody (upper panel). Immunoblotting of OLA1 was also performed to corroborate the pull-down efficiency (middle panel). The input of each reaction was verified by silver staining (bottom panel).

Immunoprecipitation followed by Western blot analysis using HEK293 cells demonstrated that GSK3β co-immunoprecipitated with both endogenously expressed OLA1 and ectopically expressed FLAG-tagged OLA1, and reciprocally, endogenous OLA1 co-immunoprecipitated with HA-tagged GSK3β (Figure 6A-6D). Two mutant forms of the OLA1 protein—one containing a point mutation at the G4 motif of the G domain that abolishes ATP-binding (N230A) [11], and another truncated protein with a deleted C-terminal TGS domain (∆TGS)—were also ectopically expressed and immunoprecipitated (Figure 6A). Interestingly, while deletion of the TGS domain of OLA1 abolished the co-immunoprecipitation, the N230A mutant OLA1 continued to interact with the endogenous GSK3β (Figure 6B). However, when two forms of the GSK3β proteins—the wild-type and the constitutively active form mediated by the S9A point mutation [25]—were ectopically expressed and immunoprecipitated, endogenous OLA1 was bound more abundantly with the S9A form than with the wild-type GSK-3β (Figure 6D). Together, these results suggest that OLA1 interacts with GSK3β in intact cells and the TGS domain in the OLA1 protein is required for the interaction. Furthermore, *in vitro* binding assays using the recombinant OLA1 and GSK3β proteins corroborated the direct binding of His-tagged OLA1 and GST-tagged GSK3β (Figure 6E).

## DISCUSSION

In this report, we demonstrated a positive role of OLA1 in cancer progression in lung cancer. Our IHC staining of an array of various human lung cancer specimens revealed that high expression of OLA1 was significantly correlated with advanced TNM stages and lymph node metastasis, whereas our meta-analysis using the KM plotter showed that high-level expression of OLA1 was significantly associated with poor prognosis in patients with lung cancer, especially in lung adenocarcinoma. One previous report indicated that OLA1 is overexpressed in colon, stomach, ovary, uterus, and lung cancers at the mRNA level, and in colon cancer at the protein level, compared to their normal tissue counterparts [18]. However, in a recent study from our group, based on IHC analyses of 160 cases of breast cancer, we found lower OLA1 protein expression was associated with higher risk of relapse and a decreased disease specific survival [26], indicating that OLA1 may play a cancer type-specific role in cancer progression.

In this study, we found a higher expression of OLA1 in lung squamous cell carcinoma than in other histological types among 110 lung cancer samples from a commercial tissue array. However, our meta-analysis showed that the high-expression OLA1 was significantly associated with poor OS in lung adenocarcinoma patients but not in squamous carcinoma patients. The results of OLA1 expression and prognosis analysis were from different patient cohorts, which may be the reason for the inconsistence. Further research is needed to evaluate the role of OLA1 in different histological types of lung cancer.

Interestingly, in the present study, we established a strong inverse correlation between OLA1 expression and E-cadherin protein staining for all histological types of lung cancer. Because loss of the epithelial marker E-cadherin is a characteristic of EMT [3, 5], we hypothesized that overexpression of OLA1 in lung cancer cells might cause downregulation of E-cadherin and progression of EMT, thus contributing to the acquisition of more aggressive and metastatic phenotype. In an experimental EMT system, we found that OLA1-knockdown A549 cells were more resistant to TGF-β1-induced EMT than the control-siRNA transfected cells, as assessed by the morphological changes, as well as the expression of E-cadherin. In subsequent studies, we explored possible upstream factors that mediated the effect of OLA1 on E-cadherin, including Snail, the transcriptional suppressor of E-cadherin, and GSK-3β, a kinase that phosphorylates Snail and so enables its degradation. We discovered that the underlying mechanism, by which OLA1 regulates EMT, is related to OLA1's inhibitory effect on GSK3β, and the GSK3β-mediated Snail/E-cadherin signaling. Importantly, we further found that the OLA1-mediated GSK-3β inhibition is independent of either TGFβ-Smad signaling or Akt phosphorylation but *via* a direct action on GSK3β.

By protein chemistry analysis, the OLA1/YchF protein is predicted to be a regulatory protein that interacts with downstream client protein(s) by switching between its ADP- and ATP-bound form [11, 27]. Our current study provided the first evidence to show that GSK3β is such an OLA1 client protein (binding partner). Interestingly, the C-terminal TGS domain of OLA1was found to be necessary for its binding with GSK3β. However, there is no direct information on the functions of the domain, which is shared by a few enzymes, including threonyl-tRNA synthetase, Obg family GTPases, and the bacterial guanosine polyphosphate phosphohydrolases/synthetases (SpoT/RelA) [13, 28]. Our study provides the first indication for its requirement in the function of the whole protein. Apparently, a consequence of the OLA1-GSK3β interaction is to secure the phosphorylation of the kinase at the Ser9 residue.

Under OLA1 deficiency, the site becomes under-phosphorylated and resistant to TGF-β1-induced phosphorylation. The presence of OLA1 in the GSK3β complex may: (1) facilitate the action of the upstream kinases that phosphorylate GSK3β such as Akt, or (2) prevent the de-phosphorylation of Ser9-p by protein phosphatases such as PP1 [29].

Data from the present study suggest that OLA1 has a negative effect on GSK3β activity, a positive effect on stabilization of Snail, and a negative correlation with the E-cadherin protein. These findings shed new light on understanding the specific function of OLA1 in regulating EMT. In our previous studies, OLA1 knockdown cancer cells exhibited decreased cell migration and invasiveness [17], and enhanced cell-matrix adhesion [30]. Now we realize that these phenomena may, in part, be mediated by the altered activities of GSK3β and/or Snail. It is noteworthy that OLA1 may have more diverse functions such as communicating with the upstream of the GSK3β-dependent pathway or with many unexplored pathways. The recent discovery of the binding partners of OLA1, HSP70 [16] and BRCA1 [31], strongly indicates the broad involvement of OLA1 in cellular function.

In summary, our results revealed that OLA1 is a novel endogenous suppressor of GSK3β, and through inhibiting the GSK3β/Snail/E-cadherin signaling, OLA1 positively regulates the EMT process in lung cancer cells. This study also demonstrated that high-level expression of OLA1 is associated with lymph node metastasis, advanced TNM stages, and poor prognosis in lung cancer including NSCLC. These findings might lead to the application of OLA1 as a potential prognostic marker or therapeutic target for lung cancer.

## MATERIALS AND METHODS

### Lung cancer tissue array and immunohistochemistry

We obtained the 120-case lung cancer tissue microarray containing 110 cores/cases of lung cancer and 10 cores/cases of normal lung tissue from US Biomax (Rockville, MD, # BC041115a), together with the associated gender and age information, histopathological diagnosis, TNM stage, and pathological grade. Each single tissue spot on every array slide is individually examined by certified pathologists according to WHO published standardizations of diagnosis, classification and pathological grade. Pathological re-confirmation report is generated and digital image captured. Before the tissue array are delivered to clients, standard immunohistochemistry tests are also performed to ensure the accuracy and specificity of tissue array products (http://www.biomax.us/support.php, Product QA/QC Info). To avoid the possible bias using small tissue in the tissue microarray for evaluating the OLA1 expression, we further evaluated the OLA1 expression in larger tissue sections cut from formalin-fixed paraffin-embedded tumor and paired normal lung tissues from 10 patients with lung adenocarcinoma. The protocols for collection and analysis of these tissues was approved by the Ethics Committee of Xinqiao Hospital of the Third Military Medical University, and all participants signed informed consent. For immunohistochemical staining, 5 μm-thick sections from paraffin-embedded tissue mounted on coated glass slides were deparaffinized, hydrated, and heated in 10mM sodium citrate buffer (pH 6.0) for 15 minutes in a steamer for antigen retrieval. After brief treatment with the Background Buster blocking agent (Innovex Biosciences, Richmond, CA), the slides were incubated with the diluted primary antibody [anti-OLA1 (Sigma-Aldrich, #HPA035790) 1:80; anti-E-cadherin (Abcam, #ab15148) 1:500] for 45 minutes. After washing with the Innovex washing buffer, the slides were incubated sequentially with STAT-Q secondary linking antibody (Innovex Biosciences), HRP-labeled streptavidin, and the Innovex substrate (DAB) for color development, following the manufacturer's instructions.

Expression of OLA1 and E-cadherin was evaluated independently by two experienced pathologists. Protein expression levels were determined semi-quantitatively by combining the proportion and intensity of the positively stained tumor cells. The percentage of positively stained tumor cells was scored as follows: 0 (no positive tumor cells); 1 (1-25% positive tumor cells); 2 (26-50% positive tumor cells); 3 (51-75% positive tumor cells); and 4 (76-100% positive tumor cells). Staining intensity was scored as follows: 0 (no staining); 1 (weak staining); 2 (moderate staining) and 3 (strong staining). The staining intensity score multiplied by the percentage of positive staining was used to define the expression levels of OLA1 and E-cadherin. The median value of all scores was used as the cut-off point for classification of the expression. Thus, lung cancer cases were classified into two groups: “low-expression” and “high-expression” in terms of each protein of interest. Cases with discrepancies were further reviewed by the original two pathologists and a senior pathologist simultaneously until a consensus was reached.

### Meta-analysis of the association of OLA1 expression with overall survival

A meta-analysis of OLA1 expression among 1928 lung cancer patients was performed using the Kaplan-Meier plotter online survival analysis software (www.kmplot.com) [32]. Kaplan-Meier plots were generated using the probe of OLA1 (219293_s_at). To analyze the prognostic value of the probe, the samples were split into two groups at the upper tertile of gene expression. The two patient groups were compared by the Kaplan-Meier plotter, and the hazard ratio with 95% confidence intervals (CI) and log rank P value were calculated.

### Cell culture and treatment

We obtained the following from the American Type Culture Collection: human lung adenocarcinoma cell line (A549), human breast cancer cell line (MDA-MB-231), and human cervical cancer cell line (HeLa). Cells were cultured in Dulbecco's Modified Eagle's medium (DMEM, Invitrogen, Calsbad, CA, USA) supplemented with 10% fetal bovine serum (FBS; HyClone, Thermo Scientific, Waltham, MA), 100 U/mL penicillin, and 100 μg/mL streptomycin (Invitrogen). A549 cells were starved in medium containing 0.1% FBS for 16 hours before treatment with 2.5 ng/mL TGF-β1 (R&D Systems, Minneapolis, MN).

### RNAi-mediated gene knockdown

Human OLA1 cDNA (NM_013341.3)-specific siRNA (SASI_Hs01_00244684) and the control siRNA (MISSION siRNA Universal Negative Control, #SIC001) were acquired from Sigma-Aldrich (St Louis, MO). Cells seeded in 6-well plates were transiently transfected with 5 μM siRNA with the DharmaFECT1 siRNA Transfection Reagent (Thermo Scientific) according to the manufacturer's instructions. To establish stable OLA1-knockdown cell lines, we used SMARTvector lentiviral shRNA particles (Thermo Scientific) containing a shRNA sequence specific for OLA1 (TGTTCGCTTCCAGATACTT) and the control shRNA sequence at a range of 5-20 TU/cell. Cells expressing the respective shRNAs were selected with puromycin (5μg/mL) for 1 month. The knockdown efficiency of the target gene was verified by Western blot analysis.

### Gene transfection

To achieve ectopic OLA1 expression, a cDNA fragment encoding full-length human OLA1 cDNA (NM_013341.3) was cloned into the pdEYFP-N1gen plasmid with a C-terminal YFP tag [16, 17]. Cells in 6-well plates were transfected with the OLA1-YFP plasmid (4 μg DNA/well) or the pdEYFP-N1gen (YFP control) plasmid using Lipofectamine 2000 (10 μl/well, Invitrogen). The parental plasmids were donated by Stefan Wiemann of the European Molecular Biology Laboratory [21]. To express tagged proteins for immunoprecipitation, human OLA1 cDNA was modified into sequences carrying the full open reading frame (396 aa), the N230A point mutation, or the C-terminal deletion mutation without the TGS domain (∆TGS, 304 aa), and each of them was cloned into the pIRESneo3-FLAG vector (Clontech, Mountain View, CA), allowing the expression of N-terminal FLAG-tagged wild-type or mutant OLA1 proteins. We obtained the HA-GSK3-beta-wt-pcDNA3 and HA-GSK3-beta-S9A-pcDNA3 plasmids encoding C-terminal HA-tagged wild-type proteins and the constitutively active mutant GSK3β proteins [20], respectively, from Addgene (Cambridge, MA, #14753 and #14754). HEK293 cells were transfected with these DNA constructs for 48 hours before they were harvested for immunoprecipitation.

### Immunoblot analysis

Western blot analysis was performed according to our standard procedures as described in earlier studies [16, 17]. Briefly, equal amounts of cellular protein were separated by SDS-PAGE and transferred onto polyvinylidenefluoride (PVDF) membranes (Bio-Rad, Hercules, CA). The membrane was blocked with 5% non-fat milk for 1 hour, and subsequently incubated with primary and secondary antibodies. Immunoreactive bands were visualized with a chemiluminescence detection system (Thermo Scientific Pierce, Rockford, IL). All antibodies used in these studies were purchased from Cell Signaling Technology (Danvers, MA) with the following exceptions: anti-OLA1, Abcam (Cambridge, MA); anti-β-actin, Sigma-Aldrich; anti-Snail, Thermo Scientific; and anti-rabbit (or mouse) IgG, peroxidase-linked secondary antibody, GE Healthcare (Pittsburgh, PA).

### Immunoprecipitation and *in vitro* binding assay

For IP of endogenous protein (GSK3β)and the HA-tagged proteins, HEK293 cells were lysed by IP lysis buffer (Thermo Scientific), and the protein lysate was incubated with 1μg antibody or the corresponding normal IgG(as the negative control)overnight at 4°C with gentle rotation. The mixture was then incubated with 50 μl of pre-washed protein A/G plus agarose (ThermoScientific) for 2 hours at 4°C. Immunoprecipitated complexes were collected by centrifugation at 7,000×*g* for 1 minute, and washed five times with 1 mL washing buffer. The immunoprecipitate was then eluted by incubating with 100 μl of elution buffer for 10 minutes at room temperature, and the elution was subjected to immunoblot analysis. In these experiments anti-GSK3β antibody was obtained from Cell Signaling Technology (#9315), and anti-HA was from Roche Applied Science (#14034800). For IP of FLAG-tagged proteins, anti-FLAG M2 magnetic beads (Sigma-Aldrich) were used according to the manufacturer's instructions. To verify protein-protein interaction *in vitro*, recombinant GSK3β-GST (SignalChem, Richmond, BC, Canada, #G09-10G) and PERK-GST fusion proteins (SignalChem #E11-11G) were incubated with His-tagged human OLA1 recombinant protein (custom made by Epoch Life Science, Houston, TX) in binding buffer [6.5 mM NaPO4 (pH 7.4), 140 nM NaCl, and 0.02% Tween 20%] for 1 hour at room temperature. His-OLA1 protein was precipitated using the Dynabeads His-tag isolation system (Life Technologies, Carlsbad, CA), and the precipitates were analyzed by Western blotting using anti-GST (SignalChem) and anti-OLA1 (Sigma-Aldrich) antibodies. The input of proteins used in each reaction was verified by silver staining (Thermo ScientificPierce).

### Statistical analysis

Data were presented as mean±SD of three independent experiments at least. Quantitative data between groups were compared using Student's *t*-test. Categorical data were analyzed by the chi-square test or Fisher's exact test. The correlation between OLA1 and EMT markers was analyzed using a Spearman's rank correlation test. *P* < 0.05 was considered statistically significant. All the statistical analyses were performed using SPSS version 17.0 software (SPSS, Inc., Chicago, IL, USA).
